# Associations of fear of physical activity, coping style and self-reported exercise behavior in patients with chronic heart failure

**DOI:** 10.1371/journal.pone.0309952

**Published:** 2024-09-05

**Authors:** Heike Spaderna, Vincent M. Brandenburg, Michael Lauterbach, Tara M. Partetzke, Sandra U. Schwab, Frederik Voss, Ingrid Kindermann

**Affiliations:** 1 Department of Nursing Science, Health Psychology, Trier University, Trier, Germany; 2 Department of Cardiology and Nephrology, Rhein-Maas-Klinikum, Würselen, Germany; 3 Department of Cardiology, Heart Center Trier, Krankenhaus der Barmherzigen Brueder, Trier, Germany; 4 Graduates’ Center, Trier University, Trier, Germany; 5 Department of Electrophysiology, Heart Center Trier, Krankenhaus der Barmherzigen Brueder, Trier, Germany; 6 Department of Internal Medicine III, Cardiology, Angiology and Intensive Care Medicine, University Hospital, Saarland University, Homburg Saar, Germany; Tehran University of Medical Sciences, ISLAMIC REPUBLIC OF IRAN

## Abstract

**Aims:**

Fear of physical activity (PA) is discussed as a barrier to regular exercise in patients with heart failure (HF), but HF-specific theoretical concepts are lacking. This study examined associations of fear of PA, heart-focused anxiety and trait anxiety with clinical characteristics and self-reported PA in outpatients with chronic HF. It was also investigated whether personality-related coping styles for dealing with health threats impact fear of PA via symptom perception.

**Methods and results:**

This cross-sectional study enrolled 185 HF outpatients from five hospitals (mean age 62 ± 11 years, mean ejection fraction 36.0 ± 12%, 24% women). Avoidance of PA, sports/exercise participation (yes/no) and the psychological characteristics were assessed by self-reports. Fear of PA was assessed by the Fear of Activity in Situations–Heart Failure (FActS-HF15) questionnaire. In multivariable regression analyses higher NYHA class (*b* = 0.26, *p* = 0.036) and a higher number of HF drugs including antidepressants (*b = 0*.*25*, *p* = 0.017) were independently associated with higher fear of PA, but not with heart-focused fear and trait anxiety. Of the three anxiety scores only increased fear of PA was independently associated with more avoidance behavior regarding PA (*b* = 0.45, SE = 0.06, *p* < 0.001) and with increased odds of no sports/exercise participation (OR = 1.34, 95% CI 1.03–1.74, *p* = 0.028). Attention towards cardiac symptoms and symptom distress were positively associated with fear of PA (*p* < 0.001), which explained higher fear of PA in patients with a vigilant (directing attention towards health threats) coping style (*p* = 0.004).

**Conclusions:**

Fear of PA assessed by the FActS-HF15 is a specific type of anxiety in patients with HF. Attention towards and being distressed by HF symptoms appear to play a central role in fear of PA, particularly in vigilant patients who are used to direct their attention towards health threats. These findings provide approaches for tailored interventions to reduce fear of PA and to increase PA in patients with HF.

**Trial registration:**

ClinicalTrials.gov ID: NCT02898246.

## Introduction

Heart failure (HF) is a complex clinical syndrome that results from any structural or functional impairment of the heart to fill with or to eject blood [1,2]. HF prevalence increases with age and exceeds 10% in those aged 70 years or older [1]. Although the incidence of HF remains stable, aging populations lead to increasing absolute numbers of patients with HF [2].

The role of exercise in chronic HF has changed considerably over the last decades [3] and exercise and regular physical activity (PA) have become central in the non-pharmacological treatment of both HF with reduced and with preserved ejection fraction [1,4]. However, physical activity levels in patients with HF are lower compared with persons without HF [5], and adherence to exercise recommendations is far from optimal [6,7].

Fear of PA, also termed fear of movement or kinesiophobia, has gained attention as a potential barrier to PA in patients with HF [8–12]. Research often refers to the construct of fear of movement that was originally developed in patients with chronic pain to describe their avoidance of painful movements in the context of the fear avoidance model of pain [8,13,14]. Considering that main symptoms in patients with HF are shortness of breath, exercise intolerance, fatigue and weakness, particularly in the context of physical exertion [15], the application of a model that focusses on musculoskeletal pain [16,17] is disputable.

In order to develop HF-specific interventions to reduce fear of PA, an adequate theory is warranted [18] and the meaning of fear of PA in HF needs to be established by describing its network of associations with other constructs [19]. Cross-sectional research on kinesiophobia in cardiovascular populations has reported associations of this fear with various anxiety-related measures. These include state and trait anxiety [10], anxiety symptoms experienced”during the last 7 days“, assessed by the Hospital Anxiety and Depression Scale (HADS) [20,21], generalized anxiety disorder [22], and cardiac anxiety [20,23]. Cardiac anxiety, also termed heart-focused anxiety, describes fears related to the heart which are not unique to patients with a cardiac diagnosis [24,25]. It has been emphasized that anxiety, depression, distress and personality traits interact with patients’ reporting of symptoms in HF [26]. However, studies that examine systematically associations of kinesiophobia with different forms of anxiety and symptom perception in the context of HF are lacking.

In order to fill this gap we use the Fear of Activity in Situations—Heart Failure (FActS-HF15) questionnaire, which we have developed specifically to measure kinesiophobia, i.e., fear of PA, in patients with HF. This questionnaire is based in anxiety theory and a framework of PA [10]. We consider fear of PA as a specific anxiety that is evoked in situations that require the person’s engagement in any type of PA. Anxiety-evoking situations can generally be characterized by the presence of danger stimuli and ambiguity [27]. Being physically active typically elicits symptoms such as shortness of breath and palpitations, even in healthy persons. In patients with HF these symptoms can be seen as danger stimuli. Symptoms are also ambiguous, as it might be difficult for the patient to decide whether these symptoms indicate danger for the heart or are normal side-effects of being physically active [28,29]. Consequently, PA might be appraised as a threat and the patient will experience fear.

To expand the previous validation [10,11] associations of this specific fear of PA assessed by the FActS-HF15 with anxiety measures indicative of broader anxiety conceptualizations need to be determined. In addition, associations of fear of PA with clinical characteristics, symptom distress and with avoidance of physical activity and exercise behavior in ambulatory patients with heart failure need to be described in an independent sample.

It is conceivable that personality differences in the way patients cope with threatening information such as symptom perceptions are involved in the fear process [30]. Patients with a strong personality disposition to cope with health threats by directing their attention *towards* the threat (i.e., a vigilant coping disposition) will more strongly focus on their cardiac symptoms than patients with a less vigilant personality. On the contrary, patients with a strong disposition to cope with health threats by turning their attention *away* from the threat (i.e., a cognitive avoidant coping disposition), will avoid focussing on cardiac symptoms and be less affected by them than persons with a less cognitive avoidant personality. These personality differences in how to deal with cardiac symptoms might affect patients’ fear of PA. Therefore, we also explore associations of vigilance and cognitive avoidance with fear of PA and symptom perception in HF.

The present report contributes to the validation of fear of PA as assessed by the FActS-HF15 questionnaire and to the development of theory in patients with HF. First, we examine correlations of fear of PA with heart-focused fear, and trait anxiety, and the associations of each of these anxiety scores with clinical characteristics. We expect that fear of PA is associated to disease severity, indicated by clinical characteristics (LVEF, NYHA class, NT-proBNP, ICD, number of medications), but heart-focused fear (which can exist independent of any cardiac disease) and trait anxiety are not.

Accordingly, we expect that only fear of PA, but not heart-focused fear or trait anxiety is independently associated with self-reported PA (avoidance behavior regarding physical activities, participation in exercise (other than cardiac sports group) and cardiac sports group participation).

In addition, we expect that personality differences in the way patients cope with health threats contribute to fear of PA. Patients with a more vigilant personality disposition will have higher fear of PA. This will be mediated by a stronger attention towards cardiac symptoms and higher distress experienced due to HF symptoms compared to patients with a less vigilant personality disposition. Patients with a more cognitive avoidant personality disposition will have lower fear of PA. This will be mediated by reduced attention towards cardiac symptoms and lower distress experienced due to symptoms compared to patients with a less cognitive avoidant personality.

## Methods

A multisite cross-sectional study was conducted that comprised two study arms. Study 1 applied self-report questionnaires at two measurement times, study 2 included the same self-report questionnaires and afterwards an accelerometer measurement. The study protocol was approved by the ethics committees of the State Chamber of Medicine of Rhineland-Palatinate [837.250.16 (10569)] and the Saarland [188/16] and was carried out in accordance with APA ethical principles and the Declaration of Helsinki. The study was registered with ClinicalTrials.gov, ID NCT02898246.

### Participants

Adult patients with any type of chronic HF [HF with reduced ejection fraction (HFrEF), with mildly-reduced ejection fraction (HFmrEF), as well as with preserved ejection fraction HFpEF)] [1], diagnosed by echocardiography and NT-proBNP values > 300 pg/mL without atrial fibrillation or >900 pg/mL with atrial fibrillation were eligible for the study [31,32]. Patients were excluded if they had acute myocarditis, complex ventricular arrhythmia, severe heart valve disease, unstable angina pectoris, or additional severe diseases affecting physical activity (e.g., advanced-stage cancer). Patients were also excluded if they were not able to speak German fluently or if they had any cognitive problems. To guarantee unbiased estimation of coefficients and adjusted R^2^ values in multiple linear regression analysis a minimum of two subjects per variables is sufficient [33]. We assessed in total about 50 variables, thus a minimum sample size ≥100 was considered adequate.

### Procedures

Patients of 5 different HF outpatient clinics and HF units were invited to participate in the study between October, 1, 2016 and December, 31, 2018. Patients who were hospitalized while being invited and who consented to being contacted after hospital discharge were called again four weeks after their discharge to become enrolled. This was to ensure that all participating patients were outpatients at time of recruitment. Screened patients received information about both study arms. In order to avoid that the prospect of having to wear an PA tracker might trigger fear of PA in some patients, patients were offered to choose which study arm they preferred to enter. Patients who did not want to choose were randomized to one of the two study arms. All participants gave their written informed consent.

The set of questionnaires was mailed to all participating outpatients. Participants of study arm 1 received the FActS-HF15 questionnaire a second time four weeks later. Questionnaires were returned to the study centre by regular mail. A patient who did not return the questionnaire after three reminder calls was considered a drop-out. Missing information in returned questionnaires was followed up via telephone calls. Medical parameters were provided by the collaborating sites and missing data in medical case report forms was followed-up accordingly. The present report focuses on self-reported data that was collected in both studies 1 and 2.

### Materials

Fear of physical activity was assessed with the FActS-HF15 questionnaire [10]. For each of fifteen situations of moderate to vigorous physical activity from the domains of everyday activities, leisure activities and sports patients respond to one item for emotionality (”I feel strained”) and one cognitive item (”I am worried that uncomfortable symptoms, such as breathlessness, could occur”) that together constitute anxiety [34]. Responses range from 0 (not at all) to 5 (very strong). The average score across all items yields the FActS total score with higher ratings indicating higher fear of PA. Internal consistency in the present sample was Cronbach’s α = .98 [10]. Psychometric properties were good and higher scores predicted less intensive PA such as stair climbing measured by wearable devices (accelerometers) in outpatients with HF [10].

The German version of the Cardiac Anxiety Questionnaire [CAQ, 24], the Herzangstfragebogen [HAF-17, 35], was used to assess heart-focused fear, which is not unique to the presence of heart disease. The questionnaire yields three scores. We used the subscale fear which comprises fears and worries about chest and heart sensations (8 items, e.g.,”If tests come out normal, I still worry about my heart“,”I worry that doctors do not believe my chest pain/discomfort is real”). Participants respond on a scale from 0”never”to 4”always”. The score is computed as the mean of the relative frequency ratings for each of the items in the scale [24,35]. Cronbach’s α of heart-focused fear in our sample was α = .74.

Trait anxiety and depression were assessed using the State Trait Anxiety and Depression Inventory [STADI, 36]. Participants rate 10 anxiety-related and 10 depression- related statements on a frequency scale (1”almost never”to 4”almost ever“). Responses are summed, yielding total scores between 10 and 40. Higher scores denote a stronger disposition to generally experience anxiety and depression (Cronbach’s α = .91 and .89 for trait anxiety and depression, respectively).

The coping dispositions vigilance and cognitive avoidance for physical threat were assessed by the German version of the Mainz Coping Inventory [37]. The instrument presents four scenarios of physical threat: encountering a group of persons in a dark street, driving as a passenger with an inexperienced driver in unfavorable road conditions, sitting in an airplane during a bumpy flight, visit at the dentist’s. For each scenario 5 vigilant (e.g.,”I remember previous dental treatments“) and 5 cognitive avoidant (”I think as little as possible about the upcoming treatment“) coping strategies are listed. Participants decide whether each coping strategy applies to their own way of reacting in this situation or not (0”no“, 1”yes“). Responses are summed across all situations for vigilant and cognitive avoidant reactions, respectively, yielding scores between 0 and 20 (Cronbach’s α = .81 for vigilance and α = .71 for cognitive avoidance).

To investigate whether the coping dispositions are associated with fear of PA indirectly via an increased or decreased attention towards threatening information, two mediating variables were assessed. (a) Self-reported attention towards cardiac sensations was measured via the HAF attention subscale [35]. This scale includes heart-focused attention and monitoring of cardiac activity (5 items, e.g.,”I pay attention to my heart beat”,”I check my pulse”). (b) Symptom distress was assessed by 12 heart failure-related bodily symptoms such as shortness of breath, fatigue, and swelling at the ankles. These symptoms are rated with regard to the burden caused by each stressor in the last 4 weeks (1”not at all” to 5”very much”) [10]. Both the attention and the symptom distress score are expressed as the mean value of all answered items. In the present sample Cronbach’s alpha was α = .64 for heart-focused attention and α = .83 for symptom distress.

Behavior-related outcomes included the subscale avoidance of the HAF-17 [35]. Participants state whether they avoid exercise or other physical work (German version: 4 items, e.g.,”I avoid physical exertion”). Responses assess the frequency of these experiences on a scale from 0 (”never”) to 4 (”always”), Cronbach’s alpha α = .91. Self-reported participation in a cardiac sports group and doing other sports/exercises (1 = no versus 0 = yes) was assessed.

Demographic characteristics consisted of age in years, sex, education (up to 9 years of schooling versus more than 9 years), marital status (unmarried versus married), living situation (alone versus with others), and employment (no versus yes), all coded 0 and 1, respectively. Participants also reported how informed they felt about their disease (0”not well informed“, 1”well informed“) and whether they had received any shocks in case they had an ICD. Clinical characteristics were provided by physicians (Table 1). The number of medications was documented as the number of different substance classes.

**Table 1:** Demographic, clinical, and psychological characteristics of 185 outpatients with chronic heart failure.

	*N*	*M* / *n*	(SD) / (%)	Min	Max
*Demographic characteristics*					
Age (years)	185	61.6	(11.4)	28	86
Female sex	185	45	(24.3)		
Married (yes)	184	127	(68.6)		
Living with others (yes)	185	141	(76.2)		
Education > 9 years (yes)	184	85	(45.9)		
Employment (yes)	184	51	(27.6)		
*Clinical characteristics*					
BMI (kg/m^2^)	185	28.5	(4.3)	20.4	41.6
Underlying disease	165				
Ischemic		68	(36.8)		
Idiopathic dilated		68	(36.8)		
Other		29	(15.7)		
LVEF (%)	183	36.6	(12.2)	15	75
Type of heart failure	183				
HFrEF (≤40%)		114	(61.6)		
HFmrEF (41–49%)		43	(23.2)		
HFpEF (≥50%)		26	(14.1)		
NYHA class	173				
I/I-II		52	(28.1)		
II/II-III		87	(47.0)		
III/IV		34	(18.4)		
Mean arterial pressure (mm Hg)	168	90.2	(13.5)	58.7	169.7
Hospitalisation during past year (yes)	183	99	(53.5)		
Comorbidities (yes)	185	137	(74.1)		
Number of comorbidities	183	1.5	(1.3)	0	5
Diabetes mellitus (yes)	182	21	(11.5)		
Hypertension (yes)	182	11	(6.0)		
Chronic kidney disease (yes)	185	28	(15.1)		
ICD (yes)	184	146	(78.9)		
Shock experienced (yes)	184	33	(17.8)		
NT-proBNP (pg/mL)	83	1375.5	(1854.7)	39	9064
Number of medications	184	2.9	(0.8)	1	5
ACE inhibitors (yes)	179	101	(54.6)		
AT1 receptor blockers (yes)	166	55	(29.7)		
Beta blockers (yes)	183	164	(88.6)		
Diuretics (yes)	184	163	(88.1)		
Antiarrythmics (yes)	178	37	(20.0)		
Antidepressants (yes)	176	13	(7.0)		
*Psychological characteristics*					
Well informed about heart failure (yes)	184	56	(30.3)		
Vigilance (MCI, 0–20)	185	10.5	(4.6)	0	20
Cognitive avoidance (MCI, 0–20)	185	12.3	(3.6)	4	20
Fear of PA (FActS, 0–5)	185	2.16	(1.33)	0	5
Fear of PA emotionality (FActS, 0–5)	185	2.14	(1.43)	0	5
Fear of PA worry (FActS, 0–5)	185	2.18	(1.33)	0	5
Trait depression (STADI, 10–40)	185	19.2	(5.7)	10	34
Trait anxiety (STADI, 10–40)	185	19.9	(5.98)	10	34
Heart-focused fear (HAF, 0–4)	185	1.57	(0.68)	1	3.6
Attention (HAF, 0–4)	185	1.3	(0.7)	0	3
Avoidance (HAF, 0–4)	185	1.8	(1.2)	0	4
Symptom distress (1–5)	183	2.4	(0.7)	1	4.5
Cardiac sports group (yes)	185	27	(14.6)		
Other sports/exercise (yes)	184	53	(28.6)		

*Notes*. ACE, Angiotensin-converting enzyme. BMI, body mass index. HAF, Herzangstfragebogen (Cardiac Anxiety Questionnaire). HFrEF, heart failure with reduced ejection fraction, HFmrEF, heart failure with mildly reduced ejection fraction, HFpEF, heart failure with preserved ejection fraction. ICD, implanted cardioverter defibrillator. LVEF, left ventricular ejection fraction. MCI, Mainz Coping Inventory. NT-proBNP, N-terminal pro b-type natriuretic peptide. NYHA, New York Heart Association. PA, physical activity. STADI, State Trait Anxiety Depression Inventory.

### Statistical analysis

Patients’ demographic, medical, and psychological characteristics are presented with means and standard deviations for continuous and number and proportions for categorical variables. Missing values in these data are indicated in the results. Age and gender of non-participants and participants were compared using t-tests and Fisher’s exact test. To compare participants of both subsamples t-tests and chi square tests were applied as appropriate.

Bivariate correlations of the anxiety scores with clinical and other study variables were computed using Pearson coefficients. Correlation coefficients were compared using the dependent group comparison with a shared third variable [38]. To identify multivariable correlates of fear of PA and of heart-focused fear and trait anxiety respectively, separate linear regression analyses were performed. The full model at the start was identical for each dependent variable. Each model started with all variables that were bivariately correlated (*p* ≤ 0.15) with at least one of the three anxiety scores. The outcome variables avoidance of physical activity, cardiac sports group participation and other sports/exercise and the potential mediating variables symptom distress and attention towards cardiac sensations were excluded here. The backward method (sequentially removing those characteristics from the model that were the least partially correlated with the dependent variable) was chosen to find the reduced set of independent variables that best explains the data. For variables with 0.5% to 6.5% of missing values automatic mean substitution for regression analysis was applied.

To test the hypothesis that fear of PA, but not heart-focused fear and trait anxiety, is independently associated with self-reported avoidance behavior of PA, multivariable hierarchical regression analyses for each dependent PA variable were carried out. In addition to the three anxiety scores demographic, medical and psychological characteristics were considered as covariates if they correlated with either the outcome variable (*p* ≤ 0.15) or with fear of PA. Demographic characteristics were entered in the first step, clinical characteristics in the second step, psychological characteristics in the third step. Due to the high correlation of trait anxiety with trait depression, trait depression was excluded. Fear of PA was entered in the final step to test whether it contributed to the outcome above and beyond the other factors. After regressing avoidance behavior on the selected covariates plus the anxiety scores, a second, reduced model was run that only included the significant factors. To test the same hypothesis for the dichotomous variables, cardiac sports group and other sports/exercises, hierarchical logistic regression analysis was applied. Automatic mean substitution for missing values is not feasible for logistic regression. Thus, cases with missing values were omitted and NYHA class (*n* = 173) was not included.

Finally, we tested whether each of the coping dispositions vigilance and cognitive avoidance indirectly affects fear of PA via the hypothesized mediators heart-focused attention and symptom distress using the bootstrap test [39] of the PROCESS macro v4.1 for SPSS [40]. The bootstrapping procedure used 10,000 bootstrap samples and 95% confidence intervals. This was run first for vigilance and the total FActS score and rerun for cognitive avoidance. Both mediators were entered simultaneously. All analyses were conducted with SPSS, version 28. Significance tests were two-sided with *p* < 0.05.

## Results

**Fig 1:**
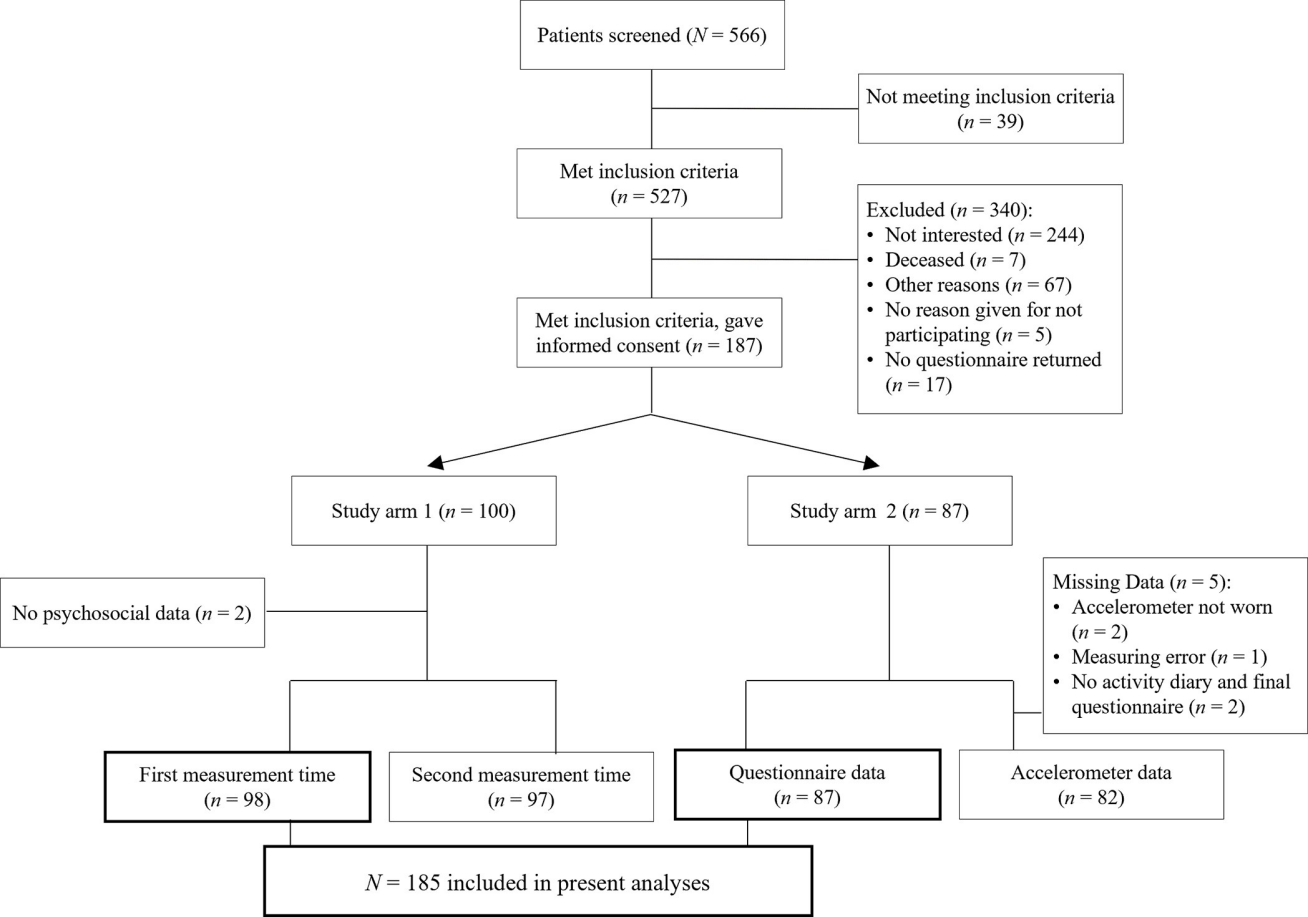
Flowchart presenting the sequence of patient enrollment including drop-outs.

In the collaborating sites 566 patients were screened between October 2016 and October 2018. Of 527 eligible patients, 187 consented to participate. The centers enrolled 20, 17, 57, 90, and 1 patient, respectively. The questionnaire data provided by 185 participants (n = 98 study 1, n = 87 study 2) were analyzed (Fig 1).

The 185 participants were younger than the 340 non-participants (*M* = 61.6, *SD* = 11.4 versus *M* = 71.1, *SD* = 13.2, *t*(428.58) = –8.58, *p* < 0.001). The lower proportion of women among participants compared to non-participants did not reach statistical significance (24% versus 32% women, Fisher’s exact test *p* = 0.071). Characteristics of participating patients are presented in Table 1. Of note, participants from both study arms were comparable regarding demographic, clinical and psychosocial data (S1 Table). All anxiety scores were significantly intercorrelated. Fear of PA accounted for only 17% in the variation of heart-focused fear, indicating that these scales measure different types of anxiety. Fear of PA accounted for 24%, heart-focused fear for 32% in the variation of trait anxiety.

### Associations of clinical characteristics with anxiety scores

Bivariate correlations of the three anxiety scores with demographic, clinical and psychological characteristics are presented in Table 2. Only higher fear of PA (especially emotionality, *r*(173) = 0.26, *p* < .001 and *r*(184) = 0.24, *p* = .001) was related to higher NYHA class and more different medications. The negative correlation of LVEF with fear of PA did not reach statistical significance but it was significantly stronger than the small positive correlations of LVEF with heart-focused anxiety (*z* = –1.82, *p* = 0.034) and with trait anxiety (*z* = –2.45, *p* = 0.007). NT-proBNP was not systematically measured at all sites and was only available in 83 patients. There was a trend for higher NT-proBNP values to be associated with higher fear of PA, particularly with emotionality (*r*(83) = .18, *p* = 0.108). NT-proBNP was unrelated to cardiac fear and trait anxiety. Neither having an ICD nor having experienced at least one shock among ICD recipients was associated with any of the anxiety scores (Table 2).

**Table 2:** Correlation coefficients of three anxiety measures (FActS-HF15, HAF Fear, Trait Anxiety) with demographic, clinical, and psychosocial characteristics in outpatients with chronic HF.

	*N*	Fear of PA in heart failure (FActS-HF15)	Heart-focused fear (HAF)	Trait Anxiety (STADI)
Age (years)	185	0.13	0.078	–0.004
Sex (male = 0, female = 1)	185	**0.208** **	0.045	0.007
Married (no = 0, yes = 1)	184	0.033	–0.006	0.068
Living with others (no = 0, yes = 1)	185	–0.004	0.024	0.001
Education > 9 years (no = 0, yes = 1)	184	–0.182 *	–0.258 **	–0.05
Employment (no = 0, yes = 1)	184	–0.228 **	–0.205 **	–0.05
BMI (kg/m^2^)	185	0.126	0.103	0.174 *
Comorbidities (no = 0, yes = 1)	185	0.177 *	0.175 *	0.223 *
Hospitalisation (no = 0, yes = 1)	183	0.183 *	0.195 **	0.108
NYHA class	173	**0.235** **	0.109	0.086
Number of medications	184	**0.224** **	0.096	0.123
LVEF (%)	183	–0.114	0.033	0.069
NT-proBNP (pg/mL)	83	0.154	0.040	0.015
Mean arterial pressure (mm Hg)	168	0.064	0.044	0.116
ICD (no = 0, yes = 1)	184	0.081	–0.039	–0.036
Shock experienced (no = 0, yes = 1)	184	–0.034	0.003	0.070
Well informed about HF (no = 0, yes = 1)	184	–0.161 *	–0.195 **	–0.212 **
Trait depression (STADI)	185	0.458 ***	0.358 ***	0.706 ***
Vigilance (MCI)	185	0.345 ***	0.374 ***	0.468 ***
Cognitive avoidance (MCI)	185	–0.084	–0.049	–0.233 **
Symptom distress	183	0.520 ***	0.432 ***	0.462 ***
Attention (HAF)	185	0.409 ***	0.486 ***	0.398 ***
Avoidance (HAF)	185	**0.661** ***	0.383 ***	0.467 ***
Cardiac sports group (no = 0, yes = 1)	185	–0.051	0.034	–0.052
Other sports/exercise (no = 0, yes = 1)	184	**–0.165** *	0.000	–0.033

*Notes*. BMI, body mass index. FActS-HF15, Fear of Activity in Situations Heart Failure questionnaire. HAF, Herzangstfragebogen (Cardiac Anxiety Questionnaire). HF, heart failure. ICD, implanted cardioverter defibrillator. LVEF, left ventricular ejection fraction. NYHA, New York Heart Association. MCI, Mainz Coping Inventory. NT-proBNP, N-terminal pro b-type natriuretic peptide. STADI, State Trait Anxiety Depression Inventory.

* p < 0.05

** *p* < 0.01

*** *p* < 0.001.

In the multivariable backward regression analysis that started with age, sex, education, employment, BMI, comorbidities, hospitalization, NYHA class, number of medications, LVEF, informed about the disease, trait depression, vigilance and cognitive avoidance, the disease specific factors NYHA class and number of medications including antidepressants were significantly associated with fear of PA, but were irrelevant for heart-focused fear (Table 3) and trait anxiety (S2 Table). The number of HF medications and antidepressants were both also separately associated with fear of PA in the multivariable analysis, but neither with any of the other two anxiety measures. Trait anxiety was only significantly associated with hospitalization, trait depression, and vigilance (S2 Table). Retest reliability of the FActS total score after 4 weeks in 97 patients was *r* = 0.803.

**Table 3:** Multivariable correlates of fear of physical activity and heart-focused fear in 185 outpatients with chronic heart failure.

	Fear of physical activity (FActS-HF15)					Heart-focused fear (HAF fear)				
	*b*	*(SE)*	*β*	*t*	*p*	*b*	*(SE)*	*β*	*t*	*p*
Female sex	0.461	(0.19)	0.15	2.41	0.017	–	–	–	–	–
Education > 9 years	–	–	–	–	–	–0.248	(0.09)	–0.18	–2.81	0.006
NYHA class	0.258	(0.12)	0.13	2.11	0.036	–	–	–	–	–
Hospitalisation	0.460	(0.16)	0.17	2.81	0.006	0.220	(0.09)	0.16	2.50	0.013
Number of medications	0.251	(0.10)	0.15	2.40	0.017	–	–	–	–	–
Trait depression	0.083	(0.01)	0.36	5.43	<0.001	0.034	(0.01)	0.28	4.11	<0.001
Vigilance	0.046	(0.02)	0.16	2.40	0.017	0.034	(0.01)	0.23	3.34	0.001
Constant	–1.237	(0.41)		–3.03	0.003	0.561	(0.18)		3.16	0.002
Summary	R^2^ = 0.344, R^2^_corr_ = 0.321, *F*(6, 178) = 15.53, *p* < 0.001					R^2^ = 0.273, R^2^_corr_ = 0.257, *F*(4, 180) = 16.89, *p* < 0.001				

*Notes*. *b*, unstandardized regression coefficient. β, standardized regression coefficient from backward linear regression analyses.

### Associations of fear of PA with self-reported physical activity-related behavior

Fear of PA was independently associated with higher scores in avoidance behavior, but heart-focused fear and trait anxiety were not (Table 4). Participation in cardiac sports groups was unrelated to the anxiety scores in bivariate analyses. Therefore, no multivariable regression analysis was run.

**Table 4:** Hierarchical regression analysis on self-reported physical activity avoidance behavior in 185 outpatients with chronic HF.

	*b*	*(SE)*	*β*	*t*	*p*	ΔR^2^	ΔF	df1	df2
*Step 1*: *Demographic characteristics*						0.034	2.15	3	181
Female sex	–0.192	(0.15)	–0.070	–1.27	0.207				
Education > 9 years	–0.021	(0.14)	–0.009	–0.15	0.880				
Employment	0.056	(0.15)	0.021	0.37	0.711				
*Step 2*: *Clinical characteristics*						0.078	3.08 *	5	176
BMI	0.023	(0.02)	0.084	1.49	0.137				
NYHA class	–0.171	(0.10)	–0.099	–1.74	0.084				
Hospitalisation	0.093	(0.13)	0.040	0.70	0.483				
Number of medications	–0.051	(0.08)	–0.035	–0.62	0.538				
Comorbidities	0.270	(0.15)	0.101	1.77	0.078				
*Step 3*: *Psychological characteristics*						0.274	15.29 ***	5	171
Heart-focused fear (HAF)	–0.014	(0.12)	–0.008	–0.11	0.913				
Trait anxiety (STADI)	0.011	(0.01)	0.057	0.78	0.438				
Informed about heart failure	–0.219	(0.14)	–0.086	–1.54	0.126				
**Symptom distress**	**0.297**	**(0.12)**	**0.175**	**2.54**	**0.012**				
Heart-focused attention	0.161	(0.11)	0.092	1.42	0.157				
***Step 4*: *Fear of PA***	**0.447**	**(0.06)**	**0.506**	**7.04**	**<0.001**	**0.138**	49.54 ***	**1**	**170**
Constant	–0.760	(0.53)		–1.43	0.153				
Summary	R^2^ = 0.525, R^2^_corr_ = 0.486, *F*(14, 170) = 13.40, *p* < 0.001								

*Notes*. BMI, Body mass index. HAF, Herzangstfragebogen. NYHA class, New York Heart Association class. PA, physical activity. STADI, State Trait Anxiety Depression Inventory. Trait depression is not included in the model due to its substantial correlation (*r* = 0.71) with trait anxiety. All regression coefficients and associated t- and p-values refer to the finale model (Step 4).

* p < 0.05

*** *p* < 0.001.

Multivariable regression on participation in other sports/exercises entering the same variables as in the analysis of avoidance behavior (except for NYHA class, which had too many missing values and therefore was exchanged for LVEF), yielded a significant association of fear of PA with sports/exercise participation (OR = 0.75, 95% CI 0.576 to 0.969, S3 Table). Each 1 unit increase in fear of PA independently multiplies the odds to report exercise/sports participation by 0.75, i.e. the odds of sports participation decrease with increasing fear of PA. A higher LVEF increased the odds to report exercise/sports participation (OR = 1.03, 95% CI 1.002 to 1.056). Heart-focused fear and trait anxiety were unrelated to this outcome (S3 Table).

### Direct and indirect associations of vigilance and cognitive avoidance with fear of PA

Bootstrap tests of mediation were adjusted for sex because each of the entered variables was significantly correlated with sex. Two participants had missing values in symptom distress, thus the *n* here was 183. Independent of sex, there was a significant total effect of vigilance on fear of PA (*b* = 0.09, SE = 0.02, t = 4.30, *p* < 0.001). This comprised a positive direct effect of vigilance on fear of PA (*b* = 0.05, SE = 0.02, t = 2.90, *p* = 0.004). The indirect effect of vigilance on fear of PA via the hypothesized mediators, heart-focused attention and symptom distress, indicated partial mediation (0.035, 95% CI 0.014 to 0.057; Table 5), with a slightly more pronounced effect of symptom distress over heart-focused attention (Table 5).

**Table 5:** Indirect effects of the coping dispositions vigilance and cognitive avoidance on fear of PA via the potential mediators heart-focused attention and symptom distress (*N* = 183).

	Fear of PA (FActS-HF15 total score)		
	*Effect*	*Boot (SE)*	*Boot 95% CI*
*Vigilance*			
Total indirect effect	0.035	(0.01)	(0.014; 0.057)
Heart-focused attention	0.014	(0.01)	(0.002; 0.030)
Symptom distress	0.020	(0.01)	(0.003; 0.040)
	Total model: R^2^ = 0.358, *F*(4, 178) = 30.16, *p* < 0.001		
*Cognitive avoidance*			
Total indirect effect	–0.029	(0.01)	(–0.059; –0.001)
Heart-focused attention	–0.009	(0.01)	(–0.023; 0.001)
Symptom distress	–0.020	(0.01)	(–0.045; 0.002)
	Total model: R^2^ = 0.331, *F*(4, 178) = 25.20, *p* < 0.001		

*Notes*. FActS-HF15, Fear of Activity in Situations—Heart Failure, PA, physical activity. CI, confidence interval. Effects are from the PROCESS bootstrap linear regression analyses.

Independent of sex, the total effect of cognitive avoidance on fear of PA was negative, but not statistically significant (*b* = –0.02, SE = 0.03, *t* = –0.75, *p* = 0.457). There was no direct effect of cognitive avoidance on fear of PA (*b* = 0.01, SE = 0.02, *t* = 0.41, *p* = 0.685). The total indirect effect via heart-focused attention and symptom distress was negative and more pronounced than the direct effect (Table 5), but only lower symptom distress tended to contribute to reduced fear of PA (Table 5).

## Discussion

To the best of our knowledge our study represents the largest and most comprehensive analysis of fear of PA, heart-focused anxiety, trait anxiety and the role of coping dispositions performed in patients with HF so far. Out of three anxiety scores only fear of PA assessed by the Fear of Activity in Situations—Heart Failure (FActS-HF15) questionnaire was associated with HF-specific clinical characteristics in patients with diagnosed chronic HF. Patients with a higher NYHA class and more HF-related medications including antidepressants (and also if considered without antidepressants) reported higher fear of PA than patients with a lower NYHA class and fewer medications. Fear of PA therefore appears to be associated with more advanced HF, whereas heart-focused fear and trait anxiety were unrelated to these HF-specific characteristics. In addition, there was only a small overlap of fear of PA with heart-focused fear (17% of shared variance) and with trait anxiety (24% of shared variance). Apparently, fear of PA is a specific type of anxiety which cannot adequately be assessed with more general anxiety questionnaires.

Moreover, only fear of PA, but not heart-focused fear or trait anxiety, was independently associated with self-reported avoidance behavior of PA and a reduced likelihood to participate in sports/exercise in these patients. This is line with previous findings obtained in outpatients with chronic HF: High fear of PA assessed by the FActS-HF15, but not trait anxiety, predicted less stair climbing measured by accelerometers [11] and was associated with a potentiation of the eye-blink reflex caused by PA-related words, an established physiological anxiety indicator [41]. Taken together these findings support our theoretical assumption that fear of PA is a specific type of anxiety that contributes to reduced engagement in PA in chronic HF and, accordingly, needs to be screened by a specific instrument such as the FActS-HF15.

Surprisingly, there was no association of any of the anxiety scores with cardiac sports group attendance. Yet, only a rather small proportion of patients (14.6%) reported to participate in a cardiac sports group. This rather disconcerting finding is in line with reports of a lacking participation in cardiac rehabilitation in this population [42]. Assessing fear of PA with the FActS-HF15 and addressing this specific anxiety might be one step towards increasing participation rates.

Patients with an ICD did neither report increased fear of PA nor increased heart-focused fear or trait anxiety. However, previous findings indicate that the association between different types of anxiety and ICD shocks is complex. E.g., baseline anxiety was unrelated to shocks [43], and only a small proportion of ICD patients had severe anxiety [44]. Moreover, any shock experience was only related to shock-specific fear, whereas general anxiety levels were associated with recent and total number of shocks [44]. Heart-focused fear decreased during the 24 months after device implantation, particularly in those patients who did not experience any shocks or anti-tachycardia pacing [25]. In the present study only 33 patients reported to have experienced any shock and we did not assess when ICDs were implanted or when the shocks occurred. More research is warranted to develop a theoretical reasoning regarding the interplay of types of anxiety and ICD shocks.

Our findings suggest that in the process of perceiving PA as a threat, an individual’s preferred way to cope with threatening information is also relevant. A vigilant coping disposition contributed to increased fear of PA both directly and indirectly. The direct path means that vigilant patients generally experienced higher fear of PA than less vigilant patients. The indirect path means that vigilant patients also more often pay attention to their cardiac activity and related symptoms and are more distressed by HF-related symptoms than non-vigilant patients. Increased symptom distress and heart-focused attention then in turn increase fear of PA. This supports the proposed role of cardiac symptom perception and negative appraisal of such symptoms for high fear of PA, particularly in patients with a vigilant coping disposition. Unexpectedly, higher cognitive avoidance was independent of heart-focused attention and symptom distress. Nevertheless, the expected total indirect effect [39] of cognitive avoidance on fear of PA via decreased symptom distress was evident, albeit weak. This deserves more attention in future studies.

### Clinical implications

From a clinical perspective, systematic efforts to reduce fear of PA, which was stable across 4 weeks in this sample, are crucial in order to increase patients’ engagement in daily physical activities and regular exercise. Regular PA improves central hemodynamics, endothelial function, skeletal muscle function and consequently symptoms [45]. Thus, we provide a promising approach for the development of interventions that focus on patients’ symptom perception and reappraisal during PA to reduce fear of PA, particularly in patients with a strong vigilant coping disposition.

Primary care physicians who advise patients with HF to increase their PA might pay attention to a patient’s way of coping with symptoms and of reporting worries and negative affect associated with engaging in exercise and PA. If these indicators of increased fear of PA are present, patients might benefit from an individualized approach that combines exercise training with education about which normal symptoms to expect during exercise. Physicians can offer reassurance about the safety of exercise when experiencing symptoms and can suggest to use distractions to direct a vigilant patient’s attention away from dysfunctional monitoring of his or her symptoms. This might support patients to develop adequate coping strategies and to increase their self-efficacy [46]. This can help to break the vicious cycle of exercise intolerance, fear, depression and avoidance of PA. Collaborations of physicians with psychologists trained in diagnosing and modifying dysfunctional cognitions and emotions might be a promising option for the future.

### Strengths and limitations

The strength of our study lies in drawing on psychological theory to examine the relevance of different types of anxiety measures and coping dispositions with PA reports obtained in a large multi-site study of patients with chronic HF. We provide data that demonstrates that different anxiety measures cannot be used interchangeably in order to describe fear of PA as a barrier to engaging in exercise and PA. Thus, associations between PA and different anxiety scores need to be carefully interpreted in light of the underlying theoretical concept of anxiety. Associated characteristics such as patients’ dispositions to cope with fear of PA and symptoms also need to be taken into account to derive clinical implications to reduce fear of PA and increase PA level in patients with HF.

One limitation is that these cross-sectional data preclude causal interpretations, and the test of mediation was not based on repeated measurements. However, the inferred relationship that a more vigilant coping disposition may affect symptom perception which in turn may affect fear of PA are based on a theory of personality dispositions that per definition precede actual emotional experiences and coping efforts. The fact that vigilance not only contributed to fear of PA via the mediators heart-focused attention and symptom distress, but also contributed independently of these mediators, indicates that these mediators did not completely describe the processes involved in the relationship between vigilance and fear of PA [39]. Clearly, vigilant strategies of information search directed at external sources of information, e.g., via the internet or one’s physician, or additional internal strategies such as searching one’s memory for similar past situations and their outcomes were not included here [27]. Also, the HAF attention score was less reliable (α = .64) in this outpatient sample compared to a study that assessed patients with heart failure at hospital admission [25]. Thus, these mechanisms deserve to be elucidated in future experimental and longitudinal studies.

Participants were offered the option to decided in which study arm to participate. Importantly, both clinical and psychological characteristics did not differ between both groups of patients and there was no indication of a selection bias.

NT-proBNP as an important heart failure specific clinical indicator was not systematically assessed in the participating hospitals and therefore the statistical power to detect associations with fear of PA was limited. LVEF on the other hand is less informative in HFpEF, which was present in 26 patients. Future studies might benefit from systematically assessing clinical indicators of disease severity, including ICD data on shocks.

Our data were assessed between 2016 and 2018. Since then some HF management strategies might have changed in clinical practice. However, PA levels appear to be unaffected by new medical treatments [47]. Thus, barriers to PA such as fear of PA and associated factors still deserve more attention as also stated in the new scientific statement on supervised exercise treatment in HFpEF [48].

Finally, women comprised only a quarter of the participants, although patients with HFpEF were also enrolled. A slightly higher proportion of women were excluded, but this difference was not statistically significant. More efforts are needed to enroll women with HF in future studies, in order to examine potential differences in barriers and facilitators of engagement in PA.

## Conclusions

This multisite study supports fear of PA assessed by the FActS-HF15 questionnaire as a specific type of anxiety in patients with chronic HF. The instrument appears useful to screen for fear of PA. This is recommended early in the disease process, since fear of PA appears to increase with more severe disease. Higher fear of PA is independently associated with more self-reported avoidance of PA and with a lower likelihood of exercise activities. This is in part mediated by a vigilant personality disposition based on which patients direct their attention towards ambiguous signs and symptoms that are elicited when being physically active. Thus, in order to avoid a vicious cycle of exercise intolerance, fear of PA, avoidance of PA, deconditioning, increasing fear and even less engagement in PA, early interventions are needed. These ought to take into account patients’ preferred coping style regarding threatening information.

## Supporting information

S1 Table Comparison of demographic, clinical, and psychological characteristics of outpatients with chronic HF in study arms 1 and 2. ACE, Angiotensin-converting enzyme. BMI, body mass index. HAF, Herzangstfragebogen (Cardiac Anxiety Questionnaire). HFrEF, heart failure with reduced ejection fraction, HFmrEF, heart failure with mildly reduced ejection fraction, HFpEF, heart failure with preserved ejection fraction. ICD, implanted cardioverter defibrillator. LVEF, left ventricular ejection fraction. MCI, Mainz Coping Inventory. NT-proBNP, N-terminal pro b-type natriuretic peptide. NYHA, New York Heart Association. PA, physical activity. STADI, State Trait Anxiety Depression Inventory.
(DOCX)

S2 Table Multivariable correlates of trait anxiety in 185 outpatients with chronic heart failure. *Notes*. *b*, unstandardized regression coefficient. β, standardized regression coefficient from backward linear regression analyses. The analysis started with the following variables: age, sex, education, employment, BMI, comorbidities, hospitalization, NYHA class, number of medications, left ventricular ejection fraction, informed about the disease, trait depression, vigilance and cognitive avoidance (all correlated with at least one of the three anxiety scores with *p* ≤ 0.15).
(DOCX)

S3 Table Hierarchical logistic regression analysis on self-reported sports participation in 177 outpatients with chronic HF. BMI, Body mass index. HAF, Herzangstfragebogen (Cardiac Anxiety Questionnaire). LVEF, left ventricular ejection fraction. PA, physical activity. STADI, State Trait Anxiety Depression Inventory. Trait depression is not included in the model due to its substantial correlation (*r* = 0.71) with trait anxiety. All regression coefficients and associated t- and p-values refer to the finale model (Step 4).
(DOCX)
