# Supplementary material for: Associations of fear of physical activity, coping style and self-reported exercise behavior in patients with chronic heart failure
Source: PLoS One. 2024 Sep 5;19(9):e0309952. doi: 10.1371/journal.pone.0309952 (PMC11376548; doi:10.1371/journal.pone.0309952)
Supplement: S1 Table — ACE, Angiotensin-converting enzyme. BMI, body mass index. HAF, Herzangstfragebogen (Cardiac Anxiety Questionnaire). HFrEF, heart failure with reduced ejection fraction, HFmrEF, heart failure with mildly reduced ejection fraction, HFpEF, heart failure with preserved ejection fraction. ICD, implanted cardioverter defibrillator. LVEF, left ventricular ejection fraction. MCI, Mainz Coping Inventory. NT-proBNP, N-terminal pro b-type natriuretic peptide. NYHA, New York Heart Association. PA, physical activity. STADI, State Trait Anxiety Depression Inventory. (DOCX) [file pone.0309952.s001.docx]

S1 Table. Comparison of demographic, clinical, and psychological characteristics of outpatients with chronic heart failure in study arms 1 and 2.

|  | Study arm 1 | | |  | Study arm 2 | | | *P* |
| --- | --- | --- | --- | --- | --- | --- | --- | --- |
|  | (*N* = 98) | | |  | (*N* = 87) | | |  |
|  | *N* | *M* / *n* | (*SD* / %) |  | *N* | *M* / *n* | (*SD* / %) |  |
| *Demographic characteristics* |  |  |  |  |  |  |  |  |
| Age (years) | 98 | 61.7 | (11.5) |  | 87 | 61.5 | (11.3) | 0.933 |
| Female sex | 98 | 24 | 24.5% |  | 87 | 21 | 24.3% | 1.000 |
| Married (yes) | 98 | 64 | 65.3% |  | 86 | 63 | 73.3% | 0.267 |
| Living with others (yes) | 98 | 71 | 72.4% |  | 87 | 70 | 80.5% | 0.228 |
| Education > 9 years (yes) | 97 | 44 | 45.4% |  | 87 | 41 | 47.1% | 0.883 |
| Employment (yes) | 97 | 24 | 24.7% |  | 87 | 27 | 31.0% | 0.410 |
| *Clinical characteristics* |  |  |  |  |  |  |  |  |
| BMI (kg/m^2^) | 98 | 28.8 | (4.4) |  | 87 | 28,1 | (4.2) | 0.270 |
| Underlying disease | 79 |  |  |  | 86 |  |  | 0.732 |
| Ischemic |  | 34 | 43.0% |  |  | 34 | 41.2% |  |
| Idiopathic dilated |  | 33 | 41.8% |  |  | 35 | 41.2% |  |
| Other |  | 12 | 15.2% |  |  | 17 | 19.8% |  |
| LVEF (%) | 97 | 35.5 | (11.0) |  | 86 | 37.9 | (13.3) | 0.180 |
| Type of heart failure | 97 |  |  |  | 86 |  |  | 0.204 |
| HFrEF (≤40%) |  | 61 | 62.9% |  |  | 53 | 61.6% |  |
| HFmrEF (41-49%) |  | 26 | 26.8% |  |  | 17 | 19.8% |  |
| HFpEF (≥50%) |  | 10 | 10.3% |  |  | 16 | 18.6% |  |
| NYHA class | 93 |  |  |  | 80 |  |  | 0.799 |
| I/I-II |  | 27 | 29.0% |  |  | 25 | 31.3% |  |
| II/II-III |  | 46 | 49.5% |  |  | 41 | 51.2% |  |
| III/IV |  | 20 | 21.5% |  |  | 14 | 17.5% |  |
| Mean arterial pressure (mm Hg) | 87 | 90.9 | (15.0) |  | 81 | 89.6 | (11.9) | 0.539 |
| Hospitalisation during past year (yes) | 96 | 49 | 51.0% |  | 87 | 50 | 57.5% | 0.458 |
| Comorbidities (yes) | 98 | 67 | 68.4% |  | 87 | 70 | 80.5% | 0.067 |
| Number of comorbidities | 96 | 1.6 | (1.4) |  | 87 | 1.4 | (1.1) | 0.333 |
| Diabetes (yes) | 95 | 13 | (13.7) |  | 87 | 8 | (9.2) | 0.365 |
| Hypertension (yes) | 95 | 9 | (9.5) |  | 87 | 2 | (2.3) | 0.060 |
| Kidney disease (yes) | 98 | 15 | (15.3) |  | 87 | 13 | (14.9) | 1.000 |
| ICD (yes) | 97 | 75 | 77.3% |  | 87 | 71 | 81.6% | 0.585 |
| Shock experienced (yes) | 97 | 17 | 17.5% |  | 87 | 16 | 18.4% | 1.000 |
| NT-proBNP (pg/mL) | 55 | 1533.5 | (2083.0) |  | 28 | 1065.1 | (1272.4) | 0.209 |
| Number of medications | 97 | 3.0 | (0.8) |  | 87 | 2.9 | (0.8) | 0.364 |
| ACE inhibitors (yes) | 94 | 53 | 56.4% |  | 85 | 48 | 56.5% | 1.000 |
| AT1 receptor blockers (yes) | 85 | 33 | 38.8% |  | 81 | 22 | 27.2% | 0.138 |
| Beta blockers (yes) | 96 | 85 | 88.5% |  | 87 | 79 | 90.8% | 0.638 |
| Diuretics (yes) | 97 | 88 | 90.7% |  | 87 | 75 | 86.2% | 0.362 |
| Antiarrythmics (yes) | 94 | 17 | 18.1% |  | 84 | 20 | 23.8% | 0.362 |
| Antidepressants (yes) | 94 | 9 | 9.6% |  | 82 | 4 | 4.9% | 0.264 |
| *Psychological characteristics* |  |  |  |  |  |  |  |  |
| Well informed about heart failure (yes) | 97 | 26 | 26.8% |  | 87 | 30 | 34.5% | 0.267 |
| Vigilance (MCI, 0 - 20) | 98 | 10.0 | (4.5) |  | 87 | 11.1 | (4.5) | 0.109 |
| Cognitive avoidance (MCI, 0 - 20) | 98 | 12.3 | (3.7) |  | 87 | 12.4 | (3.6) | 0.894 |
| Fear of PA (FActS, 0 - 5) | 98 | 2.2 | (1.3) |  | 87 | 2.1 | (1.3) | 0.630 |
| Fear of PA emotionality (FActS, 0 - 5) | 98 | 2.2 | (1.4) |  | 87 | 2.9 | (1.3) | 0.533 |
| Fear of PA worry (FActS, 0 - 5) | 98 | 2.2 | (1.5) |  | 87 | 2.2 | (1.4) | 0.761 |
| Trait depression (STADI, 10 - 40) | 98 | 19.2 | (5.6) |  | 87 | 19.1 | (5.9) | 0.582 |
| Trait anxiety (STADI, 10 - 40) | 98 | 20.0 | (6.0) |  | 87 | 19.9 | (6.0) | 0.944 |
| Heart-focused fear (HAF, 0 - 4) | 98 | 1.5 | (0.6) |  | 87 | 1.6 | (0.7) | 0.572 |
| Attention (HAF, 0 - 4) | 98 | 1.2 | (0.6) |  | 87 | 1.4 | (0.7) | 0.156 |
| Avoidance (HAF, 0 - 4) | 98 | 1.9 | (1.1) |  | 87 | 1.7 | (1.2) | 0.122 |
| Symptom distress (1 - 5) | 97 | 2.4 | (0.7) |  | 86 | 2.4 | (0.7) | 0.896 |
| Cardiac sports group (yes) | 96 | 15 | 15.6% |  | 86 | 12 | 14.0% | 0.836 |
| Other sports/exercise (yes) | 97 | 23 | 23.7% |  | 87 | 30 | 34.5% | 0.142 |

*Notes.* ACE, Angiotensin-converting enzyme. BMI, body mass index. HAF, Herzangstfragebogen (Cardiac Anxiety Questionnaire). HFrEF, heart failure with reduced ejection fraction, HFmrEF, heart failure with mildly reduced ejection fraction, HFpEF, heart failure with preserved ejection fraction. ICD, implanted cardioverter defibrillator. LVEF, left ventricular ejection fraction. MCI, Mainz Coping Inventory. NT-proBNP, N-terminal pro b-type natriuretic peptide. NYHA, New York Heart Association. PA, physical activity. STADI, State Trait Anxiety Depression Inventory.
