# Supplementary material for: Associations of fear of physical activity, coping style and self-reported exercise behavior in patients with chronic heart failure
Source: PLoS One. 2024 Sep 5;19(9):e0309952. doi: 10.1371/journal.pone.0309952 (PMC11376548; doi:10.1371/journal.pone.0309952)
Supplement: S2 Table — Notes. b, unstandardized regression coefficient. β, standardized regression coefficient from backward linear regression analyses. The analysis started with the following variables: age, sex, education, employment, BMI, comorbidities, hospitalization, NYHA class, number of medications, left ventricular ejection fraction, informed about the disease, trait depression, vigilance and cognitive avoidance (all correlated with at least one of the three anxiety scores with p ≤ 0.15). (DOCX) [file pone.0309952.s002.docx]

**S2 Table. Multivariable correlates of trait anxiety in 185 outpatients with chronic heart failure.**

|  | **Trait anxiety (STADI)** | | | | |
| --- | --- | --- | --- | --- | --- |
|  | ***b*** | ***(SE)*** | ***β*** | ***t*** | ***p*** |
| Hospitalization | 1.188 | (0.60) | 0.99 | 1.98 | 0.049 |
| Trait depression | 0.650 | (0.06) | 0.62 | 11.66 | <0.001 |
| Vigilance | 0.292 | (0.07) | 0.16 | 2.27 | <0.001 |
| Constant | 3.773 | (1.12) |  | 3.36 | <0.001 |
| Summary | R^2^ = 0.557, R^2^_corr_ = 0.550, *F*(3, 181) = 75.97, *p* < 0.001 | | | | |

*Notes*. *b,* unstandardized regression coefficient. β, standardized regression coefficient from backward linear regression analyses. The analysis started with the following variables: age, sex, education, employment, BMI, comorbidities, hospitalization, NYHA class, number of medications, left ventricular ejection fraction, informed about the disease, trait depression, vigilance and cognitive avoidance (all correlated with at least one of the three anxiety scores with *p* ≤ 0.15).
