# Supplementary material for: Associations of fear of physical activity, coping style and self-reported exercise behavior in patients with chronic heart failure
Source: PLoS One. 2024 Sep 5;19(9):e0309952. doi: 10.1371/journal.pone.0309952 (PMC11376548; doi:10.1371/journal.pone.0309952)
Supplement: S3 Table — BMI, Body mass index. HAF, Herzangstfragebogen (Cardiac Anxiety Questionnaire). LVEF, left ventricular ejection fraction. PA, physical activity. STADI, State Trait Anxiety Depression Inventory. Trait depression is not included in the model due to its substantial correlation (r = 0.71) with trait anxiety. All regression coefficients and associated t- and p-values refer to the finale model (Step 4). (DOCX) [file pone.0309952.s003.docx]

S3 Table. Hierarchical logistic regression analysis on self-reported exercise/sports participation in 177 outpatients with chronic HF.

|  | *b* | *(SE)* | *Wald* | *df* | *p* | ***χ*^2^** | df | *p* |
| --- | --- | --- | --- | --- | --- | --- | --- | --- |
| *Step 1: Demographic characteristics* | | |  |  |  | 3.49 | 3 | 0.322 |
| Female sex | –0.561 | (0.43) | 1.702 | 1 | 0.192 |  |  |  |
| Education > 9 years | –0.313 | (0.38) | 0.663 | 1 | 0.415 |  |  |  |
| Employment | 0.013 | (0.42) | 0.001 | 1 | 0.975 |  |  |  |
| *Step 2: Clinical characteristics* | | |  |  |  | 7.44 | 5 | 0.190 |
| BMI | –0.047 | (0.04) | 1.086 | 1 | 0.297 |  |  |  |
| LVEF | 0.026 | (0.01) | 2.844 | 1 | 0.092 |  |  |  |
| Hospitalisation | –0.188 | (0.38) | 0.246 | 1 | 0.620 |  |  |  |
| Number of medications | –0.052 | (0.24) | 0.047 | 1 | 0.829 |  |  |  |
| Comorbidities | 0.011 | (0.42) | 0.001 | 1 | 0.979 |  |  |  |
| *Step 3: Psychological characteristics* | | |  |  |  | 2.39 | 5 | 0.792 |
| Heart-focused fear (HAF) | 0.315 | (0.34) | 0.875 | 1 | 0.350 |  |  |  |
| Trait anxiety (STADI) | 0.016 | (0.04) | 0.150 | 1 | 0.698 |  |  |  |
| Informed about heart failure | –0.230 | (0.39) | 0.347 | 1 | 0.556 |  |  |  |
| Symptom distress | –0.046 | (0.32) | 0.021 | 1 | 0.886 |  |  |  |
| Heart-focused attention | –0.097 | (0.32) | 0.093 | 1 | 0.760 |  |  |  |
| ***Step 4: Fear of PA*** | **–0.378** | **(0.18)** | **4.223** | 1 | **0.040** | **4.02** | **1** | **0.045** |
| Constant | 0.631 | (1.68) | 0.140 | 1 | 0.708 |  |  |  |
| Summary | –2 Log Likelihood = 195.07, Nagelkerke R^2^ = 0.135, *χ*^2^(14) = 17.50, *p* = 0.231 | | | | | | |  |
| *Reduced model, N = 183* | | |  |  |  |  |  |  |
| LVEF | 0.028 | (0.01) | 4.311 | 1 | 0.038 | 1.029 | (1.002 - 1.056) |  |
| Fear of PA | –0.292 | (0.13) | 4.832 | 1 | 0.028 | 0.747 | (0.576 - 0.969) |  |
| Constant | –1.391 | (0.61) | 5.169 | 1 | 0.023 |  |  |  |
| Summary | –2 Log Likelihood = 208.21, Nagelkerke R^2^ = 0.078, *χ*^2^(2) = 10.23, *p* < 0.006 | | | | | | |  |

*Notes*. BMI, Body mass index. HAF, Herzangstfragebogen. LVEF, Left ventricular ejection fraction. PA, physical activity. STADI, State Trait Anxiety Depression Inventory. Trait depression is not included in the model due to its substantial correlation (*r* = 0.71) with trait anxiety. All regression coefficients and associated t- and p-values refer to the finale model (Step 4). Other exercise/sports participation yes = 1, no other sports participation = 0.
